# Comparative Proteomic Analysis of Non-Bleached and Bleached Fragments of the Hydrocoral *Millepora complanata* Reveals Stress Response Signatures Following the 2015–2016 ENSO Event in the Mexican Caribbean

**DOI:** 10.3390/biology14081042

**Published:** 2025-08-13

**Authors:** Esteban de Jesús Alcantar-Orozco, Víctor Hugo Hernández-Elizárraga, Jesús Eduardo Vega-Tamayo, César Ibarra-Alvarado, Juan Caballero-Pérez, Eduardo Rodríguez de San Miguel, Alejandra Rojas-Molina

**Affiliations:** 1 Posgrado en Ciencias Químico-Biológicas, Facultad de Química, Universidad Autónoma de Querétaro, Querétaro 76010, Mexico; ealcantar20@uaq.mx (E.d.J.A.-O.); hern0713@umn.edu (V.H.H.-E.); jvega24@alumnos.uaq.mx (J.E.V.-T.); 2 University of Minnesota Genomics Center, Minneapolis, MN 55455, USA; 3 Laboratorio de Investigación Química y Farmacológica de Productos Naturales, Facultad de Química, Universidad Autónoma de Querétaro, Querétaro 76010, Mexico; cibarra@uaq.mx; 4 Max Planck Institute for Immunobiology and Epigenetics, 79108 Freiburg, Germany; caballero@ie-freiburg.mpg.de; 5 Departamento de Química Analítica, Facultad de Química, Universidad Nacional Autónoma de México, Ciudad Universitaria, Mexico City 04510, Mexico; erdsmg@unam.mx

**Keywords:** *Millepora complanata*, proteomic analysis, reef building cnidarians, hydrocorals

## Abstract

The hydrocoral *Millepora complanata* (fire coral) plays a critical role in reef structure and relies on a symbiotic relationship with Symbiodiniaceae algae. Environmental stressors derived from climate change, such as UV radiation and elevated temperatures, disrupt this symbiosis, leading to bleaching and threatening reef survival. To gain insight into the thermal stress response of this reef-building hydrocoral, this study investigates the proteomic response of *M. complanata* to bleaching during the 2015–2016 El Niño event. Fragments from non-bleached and bleached colonies of the hydrocoral *M. complanata* were collected from a coral reef in the Mexican Caribbean, and proteomic extracts were analyzed using nano-liquid chromatography–tandem mass spectrometry (nano-LC-MS/MS). Uni- and multivariate analyses were applied to identify significant differences in protein abundance. A total of 52 proteins showed differential abundance, including 24 that showed increased expression and 28 whose expression decreased in bleached fragments. Differentially abundant proteins were associated with amino acid biosynthesis, carbohydrate metabolism, cytoskeleton organization, DNA repair, extracellular matrix composition, redox homeostasis, and protein modification. These molecular alterations reflect critical physiological adaptations that may influence stress sensitivity or tolerance in hydrocorals. The findings indicate that heat stress induces molecular responses involving protein refolding, enhanced vesicular transport, cytoskeletal reorganization, and modulation of redox activity. This contributes to a deeper understanding of the molecular mechanisms underlying bleaching in reef-building hydrozoans and broadens current knowledge beyond the more extensively studied anthozoan corals.

## Simple Summary

Coral reefs are essential for marine life, but they are being damaged by rising ocean temperatures linked to climate change. One important reef-building hydrocoral, named *Millepora complanata* or fire coral, depends on a symbiotic relationship with algae of the Symbiodiniaceae family to survive. When the water becomes too warm, this partnership breaks down, causing the hydrocoral to lose its color in a process known as bleaching. To better understand how this cnidarian responds to heat stress, we studied both healthy (non-bleached) and bleached fire coral fragments collected from the Mexican Caribbean during a particularly strong El Niño event. By comparing the types and amounts of proteins between these two conditions, we found changes in 52 proteins that help the hydrocoral with energy production, maintaining its structure, repairing damage, and controlling stress responses. These results show that fire corals activate many different protective processes when stressed by heat. The distinct molecular profiles observed also suggest that *M. complanata* could serve as a useful model for studying thermal stress responses in reef-building corals. Learning about these molecular responses can help scientists develop strategies to protect coral reefs, which are vital for marine biodiversity and for the communities that depend on them.

## 1. Introduction

Cnidarians, such as scleractinian corals (Anthozoa) and hydrocorals (Hydrozoa), are responsible for the formation and maintenance of coral reefs, which are ecosystems that play an essential role in maintaining oceanic biodiversity and supporting human communities [1,2,3,4]. A key aspect of this ecological success lies in their mutualistic relationship with photosynthetic algae from the Symbiodiniaceae family. This symbiosis is fundamental for coral energy acquisition and skeletal development, as the algae provide over 95% of their photosynthetically fixed carbon to the host, facilitating calcium carbonate deposition and reef accretion [5,6,7]. However, this partnership is highly sensitive to environmental stressors associated with climate change, particularly ocean warming and acidification [8]. Such stress can disrupt the physiological equilibrium of the holobiont, impairing cellular homeostasis and triggering oxidative stress responses. A primary manifestation of this breakdown is coral bleaching, which is a process in which the accumulation of reactive oxygen species (ROS) causes the cnidarian to expel its symbiotic algae, resulting in a loss of pigmentation and exposure of the white calcium carbonate skeleton [9,10]. This disruption not only affects individual colonies but also contributes to broader declines in coral reef resilience. Climate-driven disturbances have severely impacted marine ecosystem resilience [11], and widespread bleaching events have resulted in significant coral mortality and rapid reef degradation worldwide [12]. According to the National Oceanic and Atmospheric Administration (NOAA), 2014–2017 were among the warmest years recorded in the Earth’s history. During this period of time, the third global-scale bleaching event was registered, affecting more than 75% of the coral reefs around the world, 30% of which suffered mortality [13]. Alarmingly, the frequency and severity of these events continue to rise. By 2080, coral bleaching is expected to commence in spring, overlapping with critical stages of the coral life cycle and increasing the risk of long-term population decline [14].

While several studies have explored the impact of elevated temperatures on reef-forming cnidarians using omics approaches, most of this research has focused on anthozoan species. Genomic and transcriptomic analyses have revealed that thermal stress modulates the expression of genes involved in diverse cellular processes. These include pathways related to growth regulation, protein homeostasis, metabolic reprogramming, and stress adaptation mechanisms. Specifically, changes have been observed in genes regulating growth arrest, chaperone activity, nucleic acid stabilization, macromolecule degradation, energy metabolism, oxidative stress response, calcium signaling, cytoskeletal dynamics, protein synthesis, apoptosis, phagocytic processes, and immune function [15,16,17,18,19,20,21,22,23]. In contrast, proteomic studies investigating the effects of thermal stress remain limited and have been primarily conducted on anthozoans such as *Acropora microphthalma* [24], *Acropora palmata* [25], *Aiptasia* spp. [26], *Pocillopora acuta* [27], *Seriatopora hystrix* [28], *Acropora millepora* [29], *Montipora capitata* [30], *Orbicella faveolata* [31,32,33], and *Acropora hyacinthus* [34]. Although some omics studies on *M. complanata* have recently emerged, further proteomic research is needed to enhance our understanding of its molecular response to natural thermal stress. This study contributes to that effort by providing a comparative analysis of bleached and non-bleached *M. complanata* fragments collected during a large-scale bleaching event.

Regarding reef-forming hydrozoans, two previous studies demonstrated bleaching-related changes in the abundance of soluble proteins extracted from *Millepora alcicornis* and *M. complanata* exposed to the 2015–2016 El Niño–Southern Oscillation in the Mexican Caribbean. A differential abundance of proteins related to exocytosis, calcium homeostasis, cytoskeleton organization, and toxins was detected in *M. alcicornis* [35]. Similarly, differentially abundant proteins involved in key cellular processes, such as glycolysis, DNA repair, stress response, calcium homeostasis, exocytosis, and cytoskeleton organization, were found in bleached *M. complanata* [36]. Although these studies have provided valuable insights into the effects of bleaching on hydrocorals, it is important to further investigate the impact of elevated water temperatures on the proteome of these organisms in order to gain a deeper understanding of the molecular processes involved in the heat stress response of reef-forming hydrozoans. In this study, we conducted a comparative proteomic analysis of bleached and non-bleached *M. complanata* specimens collected after the 2015–2016 El Niño–Southern Oscillation (ENSO) using liquid chromatography–tandem mass spectrometry (LC–MS/MS). Our aim was to provide new insights into the molecular phenotype changes in hydrocorals induced by the disruption of the *Millepora–Symbiodiniaceae* symbiosis.

## 2. Materials and Methods

### 2.1. Sample Collection

Non-bleached and bleached *M. complanata* fragments were collected during SCUBA dives using a chisel and hammer in the Parque Nacional Arrecife de Puerto Morelos, Quintana Roo, México (21°00′ N, 86°460′ W), in the area known as “La Bocana Chica”, on 25 November 2016 at 10:00 A.M., 5 months after the 2015–2016 ENSO event. Hydrocoral fragments were retrieved by divers from two different colonies at a depth of approximately 4 to 5 m: one colony provided ten fragments of non-bleached hydrocoral, and the other provided ten fragments of bleached hydrocoral. Due to permit restrictions and the logistical challenges of fieldwork in marine protected areas, sampling was limited to one colony per condition. Immediately after collection, samples were frozen in liquid nitrogen and kept at ultra-low temperatures until their processing at the Autonomous University of Querétaro, in Querétaro, Mexico. The bleaching condition of the colonies was later confirmed based on chlorophyll *a* and *c_2_* content, as reported by Hernández-Elizárraga et al. [37]. Sea-water temperature records for November 2016 and previous years were obtained from https://seatemperature.info (accessed on 10 August 2025) (data recollection method: satellite-based remote sensing in combination with in situ data) [37]. Although previous proteomic studies have been conducted using a single colony [24,38], future research should aim to include multiple colonies per condition to better account for biological variability and enhance statistical robustness.

### 2.2. Proteome Extraction from Non-Bleached and Bleached Fragments of M. complanata Hydrocoral

Ten non-bleached hydrocoral fragments and ten bleached fragments of ≈10 g were placed in a sterile Petri dish at 4 °C. Subsequently, 5 mL of Tris-HCl extraction solution (50 mM Tris, 0.1 M NaCl, 5 mM EDTA, 20 mM β-mercaptoethanol; pH = 7.5) was added to each fragment and the surfaces were scraped using a Dremel Engraver glass scratcher [39]. The extracts obtained were collected in the Petri dish and then transferred to Falcon tubes for centrifugation at 10,000× *g* for 40 min at 4 °C to precipitate the excess CaCO_3_ and the largest cell components. Afterwards, the recovered supernatants were filtered through 0.45 µm Titan 3 nylon acrodisc filters (Thermo Fisher Scientific, Waltham, MA, USA) and the filtrates were lyophilized. Then, the lyophilized samples were prepared for dialysis by resuspending them in 1 mL of distilled and deionized water, followed by 24 h of dialysis using a 3 kDa membrane. Water was changed every 6–8 h. The dialyzed solutions were lyophilized and stored at −70 °C.

### 2.3. Proteomic Analysis of Non-Bleached and Bleached Extracts of M. complanata LC-MS/MS

Proteome extracts from non-bleached and bleached *M. complanata* fragments were resuspended in 0.1% RapiGest SF (Waters Corporation, Milford, MA, USA) dissolved in 50 mM ammonium bicarbonate buffer (Sigma-Aldrich, St. Louis, MO, USA). Subsequently, the samples were reduced with 10 mM dithiothreitol (Sigma-Aldrich, St. Louis, MO, USA) for 45 min at 60 °C and then alkylated with 55 mM iodoacetamide (Sigma-Aldrich, St. Louis, MO, USA) for 30 min at room temperature in the dark. Protein digestion was performed overnight at 37 °C using Trypsin Mass Spec Grade (Promega Corporation, Madison, WI, USA) at a protein-to-enzyme ratio of 50:1. Afterwards, salts were removed using a 1 cc Sep-Pak C18 Vac cartridge (Waters Corporation, Milford, MA, USA), and, finally, the samples were acidified with 0.1% formic acid (FA) (Sigma-Aldrich, St. Louis, MO, USA).

### 2.4. LC-MS/MS

Following sample preparation, liquid chromatography–tandem mass spectrometry analysis was performed on a Dionex Ultimate 3000 RSLC nano system coupled to an Orbitrap Fusion Lumos Tribrid Mass Spectrometer (Thermo Fisher Scientific, Waltham, MA, USA). Approximately 1.5 µg of digested peptides was dissolved in methanol (≥99.9% GC), suitable for LC/MS (LiChrosolv, Supelco, Merck, Darmstadt, Germany), and loaded onto an in-house packed column (15 cm length, 1.9 μm inner diameter; packed with Reprosil-Pur 120 C18, Dr. Maisch HPLC GmbH, Ammerbuch, Germany). Mobile phase A consisted of 98% water (LC-MS Chromasolv, Burdick & Jackson, Honeywell, Charlotte, NC, USA); 2% acetonitrile (ACN) (≥99.9% GC), suitable for LC/MS (LiChrosolv, Supelco, Merck, Darmstadt, Germany); and 0.1% formic acid (FA) (LC-MS-grade; Thermo Scientific, Waltham, MA, USA), while mobile phase B consisted of 98% ACN, 2% water, and 0.1% FA. The peptides were eluted using a 2–60% gradient of mobile phase B over 60 min at a flow rate of 400 nL/min. The mass spectrometer was operated in data-dependent acquisition (DDA) mode, and full MS scans were acquired from 350 to 1600 *m*/*z* at a resolution of 60,000 at *m*/*z* 200.

### 2.5. Database Search and Protein Identification

Raw MS files were processed using Sequest (Thermo Fisher Scientific, Waltham, MA, USA; version IseNode in Proteome Discoverer 1.4.1.14) and X! Tandem [The GPM, thegpm.org; version X! Tandem Alanine (2017.2.1.4)]. The protein sequence database was generated by retrieving all available *Millepora* protein entries from UniProtKB using the keyword “*Millepora*”. The resulting sequences were compiled in a custom FASTA file (Supplementary Materials, uniprot-Millepora-2022.09.09-Database.FASTA) for subsequent protein identification. Sequest was set up to search uniprot-Millepora-2022.09.09-Database.FASTA, assuming trypsin digestion, and X! Tandem were set up to search for a reverse concatenated *Millepora* UniProt 2023 database and a reverse concatenated *Millepora* UniProt 2023 database, also assuming trypsin digestion. The Sequest and X! Tandem search parameters were set to a parent ion mass tolerance of 20 ppm and a fragment ion mass tolerance of 0.6 Da. Carbamidomethyl of cysteine was specified in Sequest and X! Tandem as a fixed modification. Methionine oxidation and deamidation of asparagine and glutamine were set as variable modifications in Sequest and X! Tandem, while Glu->pyro-Glu of the N-terminus, ammonia-loss of the N-terminus, and Gln->pyro-Glu of the N-terminus were specified in X! Tandem as variable modifications.

### 2.6. Protein Identification and Validation Criteria

The Scaffold software (version Scaffold_5.2.0; Proteome Software Inc., Portland, OR, USA) was used to validate MS/MS-based peptide and protein identifications. Peptide matches were accepted if they achieved a probability greater than 87.0%, corresponding to a false discovery rate (FDR) of less than 5.0%. Percolator was used to estimate the posterior error probability (the probability of incorrect peptide assignment), and peptide probabilities were assigned by the Peptide Prophet algorithm [40]. Protein matches were accepted if they could be established at a probability greater than 99.0% and if they contained at least two identified peptides. Protein probabilities were calculated by the Protein Prophet algorithm [41]. Proteins sharing similar peptides that could not be distinguished based solely on MS/MS data were grouped to ensure the most parsimonious representation of the protein list.

### 2.7. Functional Annotation and Gene Ontology Term Assignment

The functional annotation of the identified proteins was conducted using the Blast2GO software (version 6.0.3; BioBam Bioinformatics, Valencia, Spain). Protein sequences in FASTA format were analyzed with BLASTp against the NCBI nr database, applying a taxonomy filter for Cnidaria (taxa ID: 6073) and an e-value threshold of 1.0 × 10^−3^. The top 5 alignments per sequence were retained for subsequent mapping to Gene Ontology (GO) terms. Annotation parameters included an annotation cutoff score of 55, a GO weight of 5, a taxonomy filter for Cnidaria, an e-value hit filter of 1.0 × 10^−6^, and a maximum of 500 hits per sequence. GO terms were classified into biological processes, molecular functions, and cellular components, providing insights into the functional roles of the identified proteins in the context of coral bleaching.

### 2.8. Statistical Analysis

A spectral counting method to infer protein abundance was employed, where the number of identified MS/MS spectra directly correlates with a protein’s relative abundance. To ensure accuracy and reliability, the spectral counting data underwent normalization and statistical treatment [42,43]. Due to the data’s uneven distribution, a generalized log transformation function (glog(y) = log(y + sqrt(y^2^ + lambda)) was applied, with λ set to 1 × 10^−9^ for optimal fit. The analysis involved both univariate and multivariate approaches. Univariate analyses included Fisher’s exact test with a Bonferroni correction for multiple testing and permutation tests. Multivariate analyses consisted of pairwise correlation analysis, principal component analysis (PCA), and partial least-squares discriminant analysis (PLS-DA). Cross-validation of multivariate analysis was performed using the venetian blinds method. Variable Importance in Projection (VIP) scores were used to assess the relative influence of latent variables and identify significant biomarkers. Fold changes, expressed as log2 values, were calculated to quantify protein abundance changes. Univariate statistical analyses were conducted in RStudio 2022.07.2 (RStudio, PBC, Boston, MA, USA) and MATLAB version R2019b (The MathWorks, Inc., Natick, MA, USA) using the Permutation Test extension (available at https://github.com/lrkrol/permutationTest) (accessed on 10 August 2025) (GitHub, San Francisco, CA, USA). Chemometric analyses (PCA and PLS-DA) were performed using PLS-Toolbox 9.0 (Eigenvector Research, Inc., Manson, WA, USA). Network analysis, illustrating relationships among significant proteins, was performed using Cytoscape 3.10.1 software (Cytoscape Consortium, San Francisco, CA, USA).

## 3. Results

### 3.1. Protein Quantification and Environmental Context of the Bleaching Event

After sample collection, a proteomic analysis was conducted on ten fragments from each condition. Non-bleached fragments exhibited an average protein concentration of 38.96 ± 0.84 µg/mL, whereas bleached hydrocorals had an average protein concentration of 38.01 ± 1.37 µg/mL. Based on information from NOAA’s Coral Reef Watch satellite monitoring, the Mexican Caribbean experienced a level 1 alert for thermal stress between August and November 2016, with a 60% likelihood of coral bleaching (sources: https://coralreefwatch.noaa.gov/index.php; https://seatemperature.info) (accessed on 10 August 2025) [37]. Moreover, Hernández-Elizárraga et al. [37] reported significant reductions in chlorophyll *a* and *c_2_* concentrations in bleached fragments of *M. complanata*, consistent with the visual bleaching observed during that event.

### 3.2. Proteomic Profiling of M. complanata Under Bleaching Conditions

Following sample preparation, liquid chromatography–tandem mass spectrometry (LC-MS) analysis was employed to compare the proteomes of bleached and non-bleached *M. complanata* fragments. A total of 1030 proteins were initially identified. After filtering out those that did not meet the decoy criteria, 639 proteins remained. Subsequently, to further refine the dataset and eliminate potentially false-positive peptide identifications, a 5% FDR threshold was applied, resulting in 102 biologically relevant proteins. These 102 biologically relevant proteins were selected for subsequent statistical analyses to identify proteins with differential abundance between the two conditions. These proteins spanned a broad range of molecular weights, ranging from 9 to 261 kDa, in the proteomes of non-bleached and bleached hydrocorals (Supplementary Table S1).

### 3.3. Statistical Analysis of Spectral Count Data

The strategy employed to identify proteins with significantly different abundances—aimed at understanding the molecular response of *M. complanata* to bleaching, as well as the relationships and correlations among these proteins—combined both univariate and multivariate statistical methods.

#### 3.3.1. Univariate Analyses

Of the 102 proteins evaluated using Fisher’s exact test (Supplementary Table S1), 26 had an adjusted *p*-value below 0.05 and 41 had an adjusted *p*-value below 0.10. Therefore, a relaxed threshold of *p* < 0.10 was applied to enhance statistical power under conditions of high correlation, as commonly practiced in exploratory proteomic studies [44,45]. However, a complementary criterion for final protein selection, based on variable importance in projection (VIP) values from the multivariate analysis, was also considered, as described below. The results of the permutation tests used to assess differences in means and effect sizes are presented in Table 1. Notably, the results showed strong consistency with observed fold changes.

#### 3.3.2. Multivariate Analyses

Pairwise correlation analysis explored the relationships between proteins, revealing predominantly significant positive correlations, along with some negative ones, across 5151 comparisons among the 52 selected proteins. A summary of the results is presented in Supplementary Figure S1. Principal component analysis (PCA) identified independent sources of variation in the dataset. A two-component model explained 84.80% of the total variability, with no outliers detected. Although some separation between bleached and non-bleached groups was observed, complete distinction was not achieved (Supplementary Figure S2).

**Figure 1:**
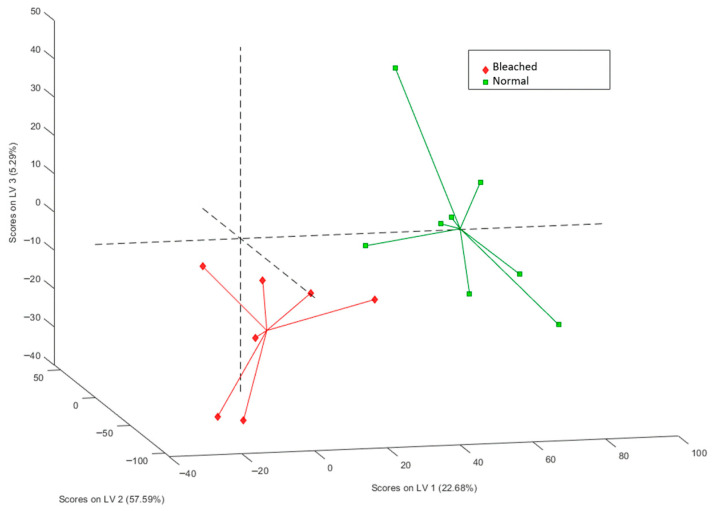
Score plot of a three-PLS-DA latent variable model showing the distinction between both groups of samples.

Partial least-squares discriminant analysis (PLS-DA) enabled the selection of a three-latent variable model that explained 85.56% of protein expression variation and 81.56% of classification category variation (Figure 1). This model exhibited clear separation between the two sample groups and demonstrated robustness, as confirmed by receiver operating characteristic (ROC) curve analysis, with area under the curve (AUC) values of 1.0000 for validation and 0.8750 for cross-validation. Sensitivity and specificity reached 1.000 during calibration, while cross-validation values were 0.714 and 0.875, respectively.

Thirty-five proteins with variable importance in projection (VIP) scores greater than 1 were selected, following standard practice (Supplementary Figure S3; Soft Independent Modeling of Class Analogy, SIMCA, Umetrics, Umeå, Sweden) [46]. As not all proteins with significant differences in univariate tests had VIP scores > 1, this study highlights the complementary nature of univariate and multivariate methods. While univariate analyses assess independent changes, multivariate methods examine interrelationships and complementary patterns among variables involved in biological processes. Therefore, combining both approaches is recommended to obtain robust insights [47]. Comparison of the protein sets identified by both methods revealed 16 overlapping proteins with adjusted *p* < 0.05 in Fisher’s exact test (45.70% of the multivariate selection) and 23 with adjusted *p* < 0.10 (65.70%). By integrating proteins with VIP > 1 and those with adjusted *p* < 0.10, a final set of 52 proteins was established, thereby enhancing the reliability of the findings. This integrated approach enabled robust identification of key proteins involved in the hydrocoral’s response to bleaching, considering both individual protein changes and their complex interactions, as illustrated in the network analysis (Supplementary Figure S4 and Supplementary Table S2).

### 3.4. Functional Annotation and Gene Ontology Term Assignment

The functional annotation and Gene Ontology (GO) analysis of the 102 proteins provided insights into their biological roles. Key visualizations summarize the GO mapping distribution, sequence similarity distribution, and combined GO annotations for biological process, molecular function, and cellular component.

**Figure 2:**
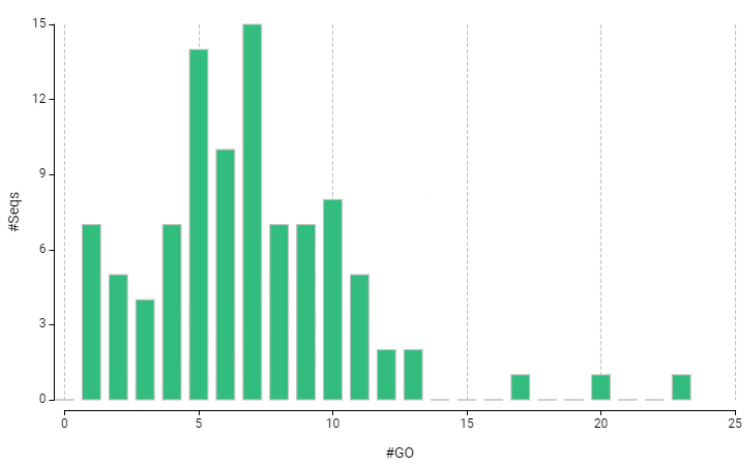
GO mapping distribution histogram.

Figure 2 presents a histogram showing the distribution of the number of GO terms mapped per protein. The *x*-axis indicates the number of GO terms assigned to each protein, while the *y*-axis shows the frequency of proteins within each category. The distribution highlights the diversity of functional annotations, with most proteins being associated with multiple GO terms. On average, each protein was annotated with 5.03 GO terms, and the median was 5, indicating a relatively high degree of functional information across the dataset. These results support the relevance of the identified proteins to various biological processes, cellular components, and molecular functions (Figure 2).

In the biological process category, several proteins were associated with cellular processes. Protein disulfide isomerase, heat shock protein 70, and peptidyl-prolyl cis-trans isomerase were annotated with the term “protein folding” (GO:0006457), reflecting their roles in the heat stress response and in maintaining redox homeostasis.

In the molecular function category, proteins such as tubulin alpha chain, tubulin beta chain, and cell division control protein 42 homolog were associated with “GTP binding” (GO:0005525).

In the cellular component category, proteins including fibrocystin-L, heat shock cognate 70 kDa, and cationic amino acid transporter were localized to the “plasma membrane” (GO:0005886).

The Sequence Similarity Distribution highlights the high alignment quality (Supplementary Figure S5). Combined GO graphs illustrate hierarchical relationships: biological process emphasizes roles in metabolism and regulation (Supplementary Figure S6); molecular function highlights binding and catalytic activities (Supplementary Figure S7); and cellular component maps subcellular localization to organelles and membranes (Supplementary Figure S8).

### 3.5. Proteins Differentially Abundant in Bleached M. complanata

The results obtained from the uni- and multivariate statistical analyses determined that a total of 52 proteins were differentially abundant when comparing both groups of hydrocorals. A summary of the average differences in protein abundance, the effect sizes of the statistical comparisons obtained by permutation tests, and fold changes are shown in Table 1, as well as their role in diverse cellular processes.

**Table 1:** Differentially abundant proteins in a bleached *M. complanata* colony identified through proteomic analysis. 1. ↑ indicates an increase in protein abundance, while ↓ indicates a decrease. These changes are directly related to the effect size. 2. Effect size values represent the magnitude of the difference in protein abundance between non-bleached and bleached fragments. 3. Fold-change values indicate the direction of differential protein abundance: positive values correspond to increased levels in bleached fragments, whereas negative values indicate decreased levels [48]. Fold-change values are log_2_-transformed; a value of ±1 represents a twofold difference in protein abundance.

No.	Protein Name	Accession Number	MW (kDa)	Protein Level ^1^	Effect Size ^2^	Fold Change ^3^
Amino acid biosynthesis						
1	Hcy-binding domain-containing protein	A0A3M6UII3	64	↑	0.8106	4.07
Carbohydrate metabolism						
2	Phosphopyruvate hydratase	A0A3M6UH98	37	↑	0.8544	3.72
Cell communication						
3	F5/8 type C domain-containing protein	A0A3M6TA43	2261	↓	−0.732	−3.83
4	PDZ domain-containing protein 2	A0A3M6TV70	403	↓	−0.4818	−3.7
Cell cycle						
5	Cell division control protein 42 homolog	A0A3M6UBV6	21	↑	0.8021	4.45
Cytoskeleton component						
6	Actin	A0A3M6UP43	42	↑	0.5179	0.18
7	Actin	A0A3M6UP81	42	↑	0.0439	0.02
8	Actin	A0A3M6UH97	56	↓	−0.3848	−0.31
9	Tubulin beta chain	A0A3M6TMY2	17	↓	−0.5705	−1.27
10	Microtubule-associated proteins 1A/1B light chain 3C-like	A0A3M6U8T6	15	↓	−0.8054	−2.49
11	Tubulin alpha chain	A0A3M6TAX7	99	↓	−0.121	−0.25
12	Myosin motor domain-containing protein	A0A3M6UZZ8	228	↑	0.5668	4.84
13	Myosin regulatory light chain ef-hand protein	A0A3M6UI41	15	↓	−0.214	−0.96
14	Actin-related protein 3	A0A3M6UDC7	44	↑	0.8317	3.25
15	Alpha-actinin, sarcomeric	A0A3M6U9E3	94	↑	0.7684	3.85
DNA repair						
16	PDDEXK_1 domain-containing protein	A0A3M6TW03	38	↓	−0.4818	−2.64
Extracellular matrix component						
17	Collagen alpha chain	B8V7R6	88	↓	−0.4818	−3.22
18	ZP domain-containing protein	A0A3M6U3D9	39	↓	−0.4818	−2.96
Protein modification and heat shock response						
19	Heat shock protein 70	Q5FB18	74	↑	0.1967	0.42
20	Ubiquitin-60S ribosomal protein L40	Q93116	15	↓	−1.1759	−1.79
21	Protein disulfide-isomerase	A0A3M6V013	37	↓	−0.5419	−1.52
22	Protein disulfide-isomerase	A0A3M6TV91	56	↓	−0.6721	−2.17
23	Heat shock 70 kDa protein cognate 5	A0A3M6U177	71	↓	−0.6242	−3.15
24	PX domain-containing protein	A0A3M6TEA6	39	↑	0.6118	2.52
25	Peptidyl-prolyl cis-trans isomerase	A0A3M6UIN0	18	↑	0.4965	3.3
26	WD repeat-containing protein 11	A0A3M6UW24	141	↓	−0.4818	−3.7
27	Calreticulin	A0A3M6TB88	51	↑	0.7684	3.35
28	UBC core domain-containing protein	A0A3M6UGY3	17	↑	0.7684	3.35
Redox homeostasis						
29	Thioredoxin domain-containing protein	A0A3M6T9K8	24	↓	−0.7799	−1.49
30	Thioredoxin-dependent peroxiredoxin	A0A3M6U7L4	22	↑	0.6057	2.26
31	Aldedh domain-containing protein	A0A3M6UUA9	58	↑	0.7784	4.35
32	Unspecific monooxygenase	A0A3M6UWA6	59	↓	−0.4818	−3.7
Signaling						
33	EF-hand domain-containing protein	A0A3M6USF3	12	↓	−0.7136	−1.7
34	EF-hand domain-containing protein	A0A3M6TLA1	14	↓	−0.6555	−1.56
35	PRKG1 interact domain-containing protein	A0A3M6TTF0	14	↓	−0.9043	−4.84
36	Serine/threonine-protein kinase TOR	A0A3M6UZI7	272	↑	0.6923	2.45
37	EGF-like domain-containing protein	A0A3M6TYF4	198	↓	−0.4818	−3.7
38	Fibrocystin-L	A0A3M6UHX3	719	↑	0.6551	5.11
39	RZ-type domain-containing protein	A0A3M6TXJ4	600	↓	−0.4818	−2.64
40	TNFR-Cys domain-containing protein	A0A3M6UKT4	56	↓	−0.4818	−3.7
Transcription						
41	Histone H4	A0A3M6UZ06	11	↓	−0.7419	−1.12
42	Histone H2B	A0A3M6THD8	14	↓	−1.0012	−2.13
43	Histone H3	A0A3M6T926	15	↓	−1.1167	−6.35
44	DUF3715 domain-containing protein	A0A3M6TH31	437	↑	0.5847	2.89
45	CCR4-NOT transcription complex subunit 1	A0A3M6T4M6	278	↑	0.668	5.94
Transport						
46	Ras-related protein Rab-1A	A0A3M6U070	25	↑	0.7684	4.35
47	Amino acid transporter	A0A3M6TNM3	181	↓	−0.4818	−3.22
48	Cationic amino acid transporter C-terminal domain-containing protein	A0A3M6UX14	96	↑	0.6237	5.62
49	Ras-related protein Rab-2A	A0A3M6UCJ4	29	↑	0.837	4.99
50	Ras-like protein 2	A0A3M6TKM7	22	↑	0.7684	3.35
Unknown						
51	Uncharacterized protein	A0A3M6V3A1	130	↓	−0.3604	−0.91
52	Uncharacterized protein	A0A3M6U169	23	↑	0.7108	3.79

## 4. Discussion

Among the differentially abundant proteins identified, one of the most biologically relevant was actin, a key cytoskeletal component consistently linked to cytoskeletal disruption and remodeling during bleaching in cnidarians [24,25,26,27,29]. Its altered abundance highlights the central role of cytoskeletal dynamics in the stress response of *M. complanata*. In addition to actin, other structural proteins such as tubulins were also affected, underscoring widespread cytoskeletal reorganization under thermal stress.

Beyond structural proteins, the 52 differentially abundant proteins identified reveal broader aspects of the cellular response to bleaching. Functional categorization showed that the most represented groups were protein modification and heat shock response (10 proteins) and cytoskeleton components (10 proteins), followed by signaling (8), transcription (5), transport (5), redox homeostasis (4), cell communication (2), extracellular matrix component (2), unknown (2), and amino acid biosynthesis, carbohydrate metabolism, DNA repair, each one with 1 protein. These categories are commonly disrupted in proteomic studies of bleaching and reflect a combination of damage and compensatory mechanisms [24,25,26,27,28,29,30,31,32,33,34]. Such functional shifts suggest that bleached hydrocorals undergo active molecular reorganization involving both injury and potential adaptation. Overall, 24 proteins were upregulated and 28 were downregulated in bleached compared to non-bleached fragments (Table 1), indicating a substantial shift in the hydrocoral proteome in response to stress. These proteins are discussed below grouped by functional category, starting with cytoskeletal components due to the central role of actin and the relevance of cytoskeletal dynamics in the bleaching response highlighted above.

### 4.1. Cytoskeleton Components

**Figure 3:**
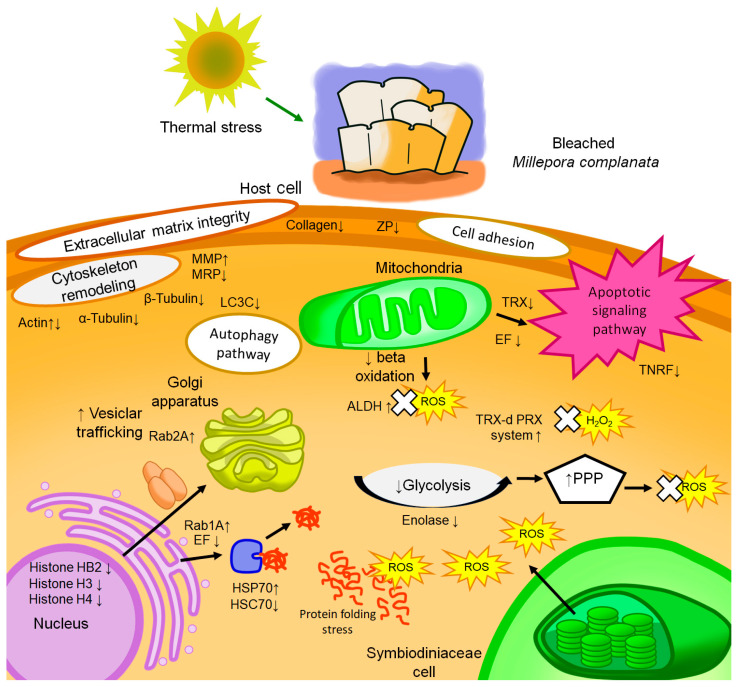
Schematic representation of cellular pathways and metabolic processes affected by the differential abundance of proteins in *M. complanata* under bleaching conditions. The figure illustrates the impact of thermal stress on host cellular functions, highlighting alterations in redox homeostasis, cytoskeletal remodeling, energy metabolism (glycolysis, pentose phosphate pathway, and beta-oxidation), vesicular trafficking, autophagy, and apoptotic signaling. ↑ indicates increased protein and processes; ↓ indicates decreased protein and processes. White crosses denote counteraction. Acronyms: thioredoxin (TRX), heat shock protein 70 (HSP70), heat shock protein 70 cognate (HSC70), reactive oxygen species (ROS), pentose phosphate pathway (PPP), aldehyde dehydrogenase (ALDH), thioredoxin-dependent peroxiredoxin (TRX-d PRX), EF-hand domain-containing protein (EF), myosin motor protein (MMP), myosin regulatory protein (MRP), microtubule-associated protein 1A/1B light chain 3C-like (LC3C), ZP domain-containing protein (ZP).

Differential abundance of actin has previously been reported in proteomic studies aimed at evaluating the effect of heat stress and bleaching on diverse anthozoan cnidarians [24,25,26,27,29]. The decreased abundance of actin found in this study is consistent with what we had previously demonstrated when comparing the soluble proteomes of bleached *M. complanata* [36] and *M. alcicornis* [35] and the transcriptomes of bleached and non-bleached *M. complanata* [36]. Actin has been recognized as a key biomarker of bleaching and heat stress [49,50], and, depending on the specific proteoform, it plays an essential role in cellular morphology, motility, and cytoskeletal organization [51,52,53,54]. The expression of actin genes is highly responsive to thermal stress in cnidarian cells, which undergo cytoskeletal reorganization in response to thermal stress and bleaching. While this reorganization has often been interpreted as a sign of cytoskeletal instability or degradation, it may also reflect a coordinated and potentially adaptive remodeling process aimed at maintaining or restoring cellular architecture during stress recovery. The removal of Symbiodiniaceae from cnidarian gastrodermal cells due to bleaching influences diverse cellular functions which depend on the actin cytoskeleton, including intracellular transport, interactions with the plasma membrane, maintenance of cell shape, and the transcriptional control of proteins associated with the cytoskeleton (Figure 3) [15,19,36,49,55,56,57,58,59,60].

Our investigation also revealed that the abundance of alpha-tubulin and beta-tubulin, which play a critical role in the formation of microtubules, was decreased in bleached hydrocorals. The decrease in the abundance of alpha- and beta-tubulin could imply a decrease in the intracellular transport of nutrients and poor cell division, which is necessary for tissue regeneration under stress conditions (Figure 3) [26]. On the other hand, the decreased abundance of Microtubule-associated protein 1A/1B light chain 3C (LC3C) suggests a reduction in autophagy under bleaching conditions. While autophagy has been proposed as a response mechanism for the elimination of endosymbionts [61,62,63,64], it has been reported that proteins associated with these processes may exhibit decreased abundances under thermal stress-induced bleaching conditions (Figure 3) [55,57].

The response of the cytoskeleton to a phenomenon such as thermal stress varies among organisms, and it has even been reported that genes encoding cytoskeletal-associated proteins can exhibit distinct differential expressions within the same study [65]. Additionally, post-translational modifications can result in the existence of multiple proteoforms, which increase proteome complexity, making proteomic analysis an even greater challenge [54,66]. This could explain the differences in the differential abundances of the other identified actin proteins. However, the observed differential abundance of cytoskeletal components in bleached hydrocorals could indicate a reorganization or disruption of the actin cytoskeleton in response to heat and oxidative stress and endosymbiont expulsion. This reorganization might be part of a broader cellular remodeling response, possibly aimed at adapting cellular structure and function to new physiological conditions during or after bleaching events (Figure 3).

### 4.2. Extracellular Matrix Component

Collagen showed a decreased differential abundance in bleached *M. complanata* fragments. Numerous transcriptomic and proteomic studies have shown differential abundance of collagen in cnidarians subjected to heat stress [17,19,20,25,35,49,55,67]. A decrease in the production of this protein could lead to loss of structural integrity and alterations in cell signaling and healing (Figure 3) [68,69]. A ZP domain-containing protein with differentially decreased abundance in bleached *M. complanata* fragments was also identified. This protein takes part in various biological processes such as fertilization and ECM formation [17,70,71,72]. Some ZP domain-containing proteins also interact with other ECM components to form stable complexes important for cellular structure and development (Figure 3) [73].

Bleaching in *M. complanata* leads to significant disruptions in cellular functions, including the stability and repair of tissues. Processes such as cell communication, regeneration, and structural organization are adversely affected. Additionally, the capacity to maintain proper cellular development and cohesion within the extracellular matrix is diminished, highlighting the extensive impact of thermal stress on fundamental biological systems.

### 4.3. Heat Shock Response

The 70-kilodalton (kDa) heat shock protein (HSP70) chaperones have been considered as molecular biomarkers to measure the physiological state of reef-forming cnidarians exposed to thermal stress [50]. In this study HSP70 was increased in bleached *M. complanata*, which is consistent with our previous study [36]. Several transcriptomic studies have analyzed the gene-expression responses to thermal stress in corals and symbiotic cnidarians [23,29,58,74,75]. The results obtained in transcriptomic investigations have also been corroborated in proteomic studies carried out to examine the heat stress response of coral holobionts [26,29,33,76,77,78]. Differential protein abundance patterns of HSP70 indicate that the modulation of this HSP is a key response of the coral holobiont to preserve protein structure and function and to stimulate cellular repair processes (Figure 3).

Thermal stress in *M. complanata* disrupts various cellular processes essential for maintaining homeostasis and coping with environmental challenges. The resulting stress affects protein folding and repair mechanisms, leading to potential dysfunctions in cell signaling, stress response, and the regulation of the cell cycle. Additionally, alterations in protein stability and degradation pathways can impair cellular repair and regeneration. These modifications reflect an adaptive yet strained cellular response to prolonged environmental stress, underscoring both the challenges faced by the holobiont and the limitations of its stress-response capacity.

### 4.4. Redox Homeostasis

Numerous studies have clearly shown that oxidative stress is closely linked to coral bleaching [79,80,81,82,83]. Thus, it is imperative for cnidarians to possess robust defense mechanisms to counteract ROS-induced damage [82,83]. The aldedh domain (increased in bleached *M. complanata*) is found in aldehyde dehydrogenase (ALDHs) enzymes. ALDHs are part of the metabolic pathway for ethanol degradation, converting toxic aldehydes into non-reactive carboxylic acids [84]. Acute thermal stress leads to a decrease in fatty acid concentration and an increase in alcohol concentration, attributed to an increase in energy reserve consumption and the generation of wax ester decomposition products, respectively [85,86]. The increased abundance of aldedh domain-containing proteins as a result of oxidative stress conditions suggests a rise in detoxification processes to counteract ROS-induced amino acid dissociation (Figure 3) [87].

Thioredoxin, which was found to be decreased in bleached hydrocorals, is related to cellular antioxidant defense mechanisms. Decreased thioredoxin reductase abundance could further compromise antioxidant defenses, suggesting an overwhelmed ROS response affecting stress-responsive protein abundance. Reduced thioredoxin activity may elevate ROS and caspase-3 activity, potentially increasing apoptosis and tissue damage in cnidarians (Figure 3) [26,33,88,89].

The increased abundance of thioredoxin-dependent peroxiredoxin found in this study is in accordance with previous reports in other cnidarians and our previous study [19,24,31,36]. An increase in the abundance of peroxiredoxin proteins suggests an enhanced capacity to reduce peroxides, which may aid in neutralizing oxidative damage, maintaining redox balance, and increasing cellular protection (Figure 3) [36].

Thermal stress induces significant alterations in redox homeostasis in *M. complanata*, contributing to oxidative damage, disrupting the balance between reactive oxygen species (ROS) production and antioxidant defenses. The resulting oxidative stress leads to increased detoxification processes to mitigate ROS damage, while simultaneously impairing antioxidant mechanisms, reducing the cell’s ability to neutralize peroxides effectively. These changes may elevate ROS levels, contributing to cellular damage, apoptosis, and compromised tissue integrity. Despite some compensatory upregulation of ROS-scavenging processes, the overall imbalance highlights the extent of oxidative damage that precedes and contributes to bleaching, as well as its broader impact on cellular stability and resilience.

### 4.5. Signaling

Two EF-hand domain-containing proteins with decreased abundance were found in bleached *M. complanata* fragments. These types of proteins are important due to their ability to bind calcium. The EF-hand domain is present in various proteins that regulate essential biological processes, mainly cellular signaling [90]. Calmodulin (an EF-hand domain-containing protein) was reported with decreased abundance in the soluble proteome of bleached *M. complanata* fragments [36]. EF-hand proteins, in general, are important for intracellular Ca^2+^ homeostasis, and this homeostasis, in turn, is crucial for proper cellular functioning. The oxidative stress produced during thermal exposure can disrupt this homeostasis, resulting in a sustained elevation of intracellular Ca^2+^ due to release from intracellular stores and entry from the extracellular environment [91,92]. Given the structural diversity of EF-hand proteins, these can exist as different proteoforms, which may exhibit distinct functional properties and contribute to the complexity of calcium regulation under stressful conditions that may ultimately culminate in bleaching [93]. The decreased abundance of two EF-hand proteins, coupled with the previous finding of decreased calmodulin abundance, could indicate that Ca^2+^-linked cellular signaling processes may be affected due to bleaching, as well as processes such as apoptosis, energy metabolism, and protein synthesis (Figure 3) [94].

Our study found that a TNFR-Cys domain-containing protein involved in apoptosis regulation [95,96] was decreased in bleached *M. complanata*. Proteins containing these domains are involved in the regulation of processes such as apoptosis [96]. The variability in TNFR expression, along with their role in apoptosis regulation, enhances stress tolerance and disease resistance in corals [23,67,97,98,99]. Therefore, this protein family emerges as a principal candidate in multiple pathways related to health and stress tolerance in these marine organisms [23,67,99]. Our findings allow us to speculate that the decreased levels of this protein in bleached fragments of *M. complanata* may indicate the inhibition of processes such as apoptosis (Figure 3).

Bleaching in *M. complanata* significantly impacts key cellular processes, including calcium homeostasis, signal transduction, and stress response pathways. Disruptions to calcium signaling may alter processes such as cell proliferation, apoptosis, and energy metabolism [90,100,101]. Changes in signaling pathways, including those associated with growth factors and kinases, suggest impaired cell growth, survival mechanisms, and stress adaptation [102,103,104]. Additionally, the regulation of apoptosis and immune responses appears compromised, potentially reducing the resilience of these organisms to environmental stressors [96,97,98,99]. These findings underscore the broad cellular disruptions caused by bleaching and the need for further research to clarify their implications.

### 4.6. Transcription

Transcriptional control undergoes significant changes in response to coral bleaching conditions, with variations in transcripts, transcription factors, and related protein expression resulting from thermal and oxidative stress [16,21,105,106]. In our study, we identified three histones linked to the transcriptional process which showed differential abundance (H4, H2B, and H3). It has been proposed that diminished levels of H4 might imply a generalized stress response that contributes to survival in adverse environments, favoring the abundance of proteins essential for stress adaptation (Figure 3) [107,108].

### 4.7. Transport

Cellular transport represents another fundamental cellular function commonly affected during bleaching events, often attributed to a rise in the response of cnidarians or an increased utilization of energy reserves [109,110,111]. Rab proteins predominantly participate in vesicle formation and transport, serving as crucial regulators of intracellular membrane trafficking [112,113,114]. Rab1A and Rab2A, proteins with increased levels in bleached hydrocorals, are involved in key cellular processes such as vesicular protein transport and migration, among others [115,116,117,118,119,120,121]. The increase in the abundance of these proteins, also linked to stress adaptation in plants [117], suggests a role in temperature stress responses and compensatory carbohydrate transport due to the loss of symbiotic algae in cnidarians, potentially influenced by changes in cytoskeletal protein abundance critical for vesicle transport (Figure 3) [16,110,111]. Bleaching in *M. complanata* disrupts intracellular transport, affecting vesicle movement, nutrient distribution, and protein trafficking. These changes reflect an increased energy demand and a response to the loss of symbiotic algae. Additionally, alterations in cytoskeletal organization may further impair transport efficiency, highlighting the extensive cellular adjustments required to cope with thermal stress and bleaching.

### 4.8. Carbohydrate Metabolism

Regarding phosphopyruvate hydratase, also referred to as enolase, which was found to be increased in bleached fragments, it is an enzyme that participates in the glycolysis pathway [122,123]. In our previous study on the soluble proteome of *M. complanata*, we observed differential abundance in two glycolysis-related proteins: alpha-enolase (upregulated) and triosephosphate isomerase (downregulated) [36]. The upregulation of alpha-enolase likely supports energy production under conditions of reduced symbiont density [36,124], whereas the downregulation of triosephosphate isomerase may suggest a metabolic shift toward the pentose phosphate pathway to mitigate oxidative stress [36,125,126,127]. However, in the present study, enolase was the only glucose metabolism-related protein exhibiting differential abundance. This discrepancy may reflect the presence of distinct proteoforms with variable expression patterns [54]. Despite the absence of other proteins showing differential abundances linked to carbohydrate metabolism, the increase in enolase observed in this study may be related to this phenomenon (Figure 3).

### 4.9. Final Considerations

Overall, the proteomic changes observed in bleached *Millepora complanata* fragments point to a widespread disruption of key cellular processes, including cytoskeletal dynamics, protein folding and modification, redox balance, signaling, and intracellular transport. These molecular alterations reflect a complex response to thermal stress, in which structural damage, oxidative stress, and loss of symbionts trigger compensatory mechanisms aimed at maintaining cellular homeostasis. The reorganization of cytoskeletal proteins, particularly actins and tubulins, suggests both disassembly and remodeling of the cytoskeleton, possibly linked to changes in vesicular transport, cell shape, and tissue integrity. Meanwhile, the upregulation of stress-related proteins, such as HSP70 and peroxiredoxins, indicates an attempt to counteract protein misfolding and oxidative damage. Despite these adaptive responses, the downregulation of antioxidant proteins and signaling regulators points to a reduced capacity to mitigate stress and maintain normal function. Taken together, these findings highlight that the bleaching response of *M. complanata* involves not only stress-induced damage but also active cellular reorganization processes that may reflect an attempt to restore physiological equilibrium in the face of environmental perturbation.

### 4.10. Limitations

This study faced several limitations, with the sampling strategy being the most relevant. Ideally, sampling multiple colonies per condition would have provided a greater genetic diversity, allowing for a more comprehensive understanding of proteomic variability and enhancing the biological relevance of the findings. However, due to the inherent challenges of working with marine organisms—such as limited accessibility and permit restrictions at the time of collection—sampling was restricted to a single colony per condition. Previous omics studies have conducted analyses employing specimens derived from single colonies or clonal laboratory stocks, thereby reducing genetic variability [16,24,38,88,128]. To mitigate this limitation and improve the robustness of the analysis, we employed a combination of univariate and multivariate statistical approaches, which have proven effective in detecting biologically meaningful patterns, even in studies with reduced sample sizes [129,130,131].

Nevertheless, we acknowledge the need for caution when interpreting the findings, as they may reflect colony-specific responses. Future research should prioritize increasing the number of biological replicates from multiple colonies to better account for inter-colony variability and improve statistical power and generalizability. In parallel, further efforts are needed to functionally validate the proteomic results and expand their biological interpretation. Although the identified proteins give us a broad overview of some of the processes that are related to bleaching in *M. complanata*, additional studies are required to evaluate protein activity and gain deeper understanding of the cellular survival processes of these holobionts. Another important limitation to consider is the lack of orthogonal validation. Future studies should aim to confirm the differential abundance of key proteins using methods such as Western blotting and the Proximity Extension Assay, which would support the proteomic findings at the protein level.

## 5. Conclusions

This study reveals proteomic alterations associated with bleaching in *M. complanata* following the 2015–2016 ENSO event, highlighting key differences in the abundance of proteins linked to stress response, cytoskeletal stability, signaling, metabolism, and cell maintenance. These changes reflect the host’s molecular adjustments to thermal stress and suggest potential mechanisms contributing to differential bleaching susceptibility.

The identified proteins are involved in critical biological processes, including redox regulation, heat shock response, intracellular transport, and extracellular matrix preservation. While these findings do not constitute direct evidence of thermal resilience, they point to physiological traits that may support short-term survival or increase vulnerability under prolonged stress.

Our results expand current knowledge of hydrocoral stress responses and contribute to the broader understanding of cnidarian bleaching mechanisms. Future studies integrating complementary omics tools, such as metabolomics and targeted proteomics, and functional assays will be key to validating these proteomic trends and clarifying their physio-logical implications. Moreover, increasing biological replication will enhance the robustness and ecological relevance of future analyses.

## Data Availability

The mass spectrometry proteomics data have been deposited with the ProteomeXchange Consortium via the PRIDE [132] partner repository with the dataset identifier PXD061020. Reviewer access details: Log in to the PRIDE website using the following details: Project accession: PXD061020; Token: j5vOh8s4Afav; Alternatively, reviewer can access the dataset by logging in to the PRIDE website using the following account details: Username: reviewer_pxd061020@ebi.ac.uk; Password: 6UYPUcmgcsuy.

## Supplementary Materials

The following supporting information can be downloaded at https://www.mdpi.com/article/10.3390/biology14081042/s1. Figure S1. Pairwise correlation analysis of the final selected protein expression data. The color bar and the size of the points stand for the magnitude and sign of the correlations. Figure S2. Score plot of a two-component PCA model showing both groups of samples. Figure S3. VIP score plot for the final classification model in the PLS-DA analysis, where the VIP values for each protein are displayed. Figure S4. Network analysis of proteomic data. Nodes are colored according to their protein levels in permutation univariate tests (shown in blue are proteins which are increased in bleached samples and shown in green are those which are decreased); their shape is related to their significance in uni- or multivariate tests (proteins significant only in the univariate test are shown with diamonds, those only in the multivariate test with squares, and those significant in both analyses with ellipses), and edges are colored according to the sign of the correlations between protein counted data (blue lines represent proteins whose levels are directly proportional and red lines those whose levels are inversely proportional). The size of the number identifying each protein is proportional to the absolute value of its effect size (Table 1). Figure S5. Sequence similarity distribution. Figure S6. Hierarchical GO classification for biological processes. Figure S7. Hierarchical GO classification for molecular functions. Figure S8. Hierarchical GO classification for cellular components. Table S1. 102 proteins evaluated using Fisher’s exact test. Table S2. Structural characterizing properties of the protein network shown in Supplementary Materials, Figure S4.
