# Supplementary material for: Comparative Proteomic Analysis of Non-Bleached and Bleached Fragments of the Hydrocoral Millepora complanata Reveals Stress Response Signatures Following the 2015–2016 ENSO Event in the Mexican Caribbean
Source: Biology (Basel). 2025 Aug 13;14(8):1042. doi: 10.3390/biology14081042 (PMC12383311; doi:10.3390/biology14081042)
Supplement: Supplementary file 1 [file biology-14-01042-s001.zip › biology-3763541-Supplementary information Figure S4 and Table S2-edited.pdf]

Comparative proteomic analysis of non-bleached and bleached fragments of the hydrocoral *Millepora complanata* reveals stress response signatures following the 2015–2016 ENSO event in the Mexican Caribbean

Esteban de Jesús Alcántar-Orozco[1], Víctor Hugo Hernández-Elizárraga[1,5], Jesús Eduardo Vega-Tamayo[1], César Ibarra-Alvarado[2], Juan Caballero-Perez[3], Eduardo Rodríguez de San Miguel[4], Alejandra Rojas-Molina[2]

1 Posgrado en Ciencias Químico-Biológicas, Facultad de Química, Universidad Autónoma de Querétaro, Querétaro, México.
2 Laboratorio de Investigación Química y Farmacológica de Productos Naturales, Facultad de Química, Universidad Autónoma de Querétaro, Querétaro, México.
3 Max Planck Institute for Immunobiology and Epigenetics, Freiburg, Germany.
4 Departamento de Química Analítica, Facultad de Química, Universidad Nacional Autónoma de México, Ciudad Universitaria, México City, México.
5 University of Minnesota Genomics Center, Minneapolis, MN, USA.
* Correspondence: Alejandra Rojas-Molina rojasa@uaq.mx

Network analysis

Network analysis in proteomics offers several advantages for understanding the complex interactions within biological systems. On the one hand, proteomic network analysis allows a holistic view of biological systems by considering not just individual proteins but also their interactions. This helps in understanding how proteins work together in pathways and networks to perform various cellular functions. On the other hand, network analysis can identify important proteins (nodes) and interactions (edges) within a network. This helps in identifying key players that have a central role in biological processes, making them potential targets or biomarkers. A final selection of 52 proteins was made based on uni- and multivariate data analyses, so that, to decrease the number of relationships, i.e., the complexity of the network, the 1326 different pairwise correlations among the 52 proteins were reduced according to their significance at the 95% confidence level. This procedure gave rise to a final set of 219 pairwise correlations (Supplementary Figure S1).

The previously discussed strong correlation structure of the data can clearly be seen, and clusters of different sizes of highly related proteins can be observed. As mentioned above, many positive correlations and some negative ones can be well noticed. These 219 significant correlations were then employed as inputs to form the edges of the network together with the 52 selected proteins which constitute the nodes. In Supplementary Figure S4 the proteomic network for the system is graphically shown, while a statistical summary of the network providing some parameters for characterizing their structural properties is reported in Supplementary Table 2. These measures provide valuable insights into their topology, connectivity, and behavior. Connected components are isolated groups of

nodes within a network that are not connected to nodes outside their group. Interestingly, among the five groups observed, the biggest one includes 43 out of 52 proteins, i.e., 82.7% of the total proteins considered, in accordance with a major source of variation determined by the first component in PCA analysis. In Supplementary Figure 5 the shapes of the nodes were additionally drawn according to their relevance in uni- or multivariate analysis, where those proteins significant only in the univariate test are shown with diamonds, those only in the multivariate test with squares, and those significant in both analyses with ellipses. As can be observed, proteins identified in the univariate test are distributed mainly within different clusters of those simultaneously identified by both methods. Meanwhile, proteins only identified in the multivariate test point toward the connected components different from the main group. Furthermore, in the network the colors of the nodes identify the changes in protein levels; in blue are shown the nodes in which they were increased, and in green are shown those in which they were decreased when going from the normal to the bleached group. As can be observed, the two levels are clearly differentiated in clusters within the main connected component and linked through the proteins marked as 2, 3, 5, and 8, which serve as a bridge between the two fully connected protein clusters. Moreover, as the size of a node's number is proportional to the absolute value of its effect size (Table 1), it can clearly be observed that proteins which exert a big effect size are identified by uni- and multivariate methods, those with a medium one by univariate methods only, and those with the smallest ones mainly by multivariate methods only. Finally, the edges marked with blue continuous lines in the network represent proteins whose levels are directly proportional, and the red lines represent those whose levels are inversely proportional; it can be seen that the direction of proportionality is in accordance with the change in the levels of the proteins.

Positive correlations often indicate that two proteins within a pathway work together to achieve a common biological function. When the abundance of one protein is increased, it may stimulate or facilitate the augmentation of the other one. This suggests that both proteins are involved in the same biological process and may act synergistically.

Proteins showing positive correlations may also have complementary functions within a pathway. This may indicate that both proteins are regulated by the same upstream factors as well. These correlations can additionally be a sign of feedback loops within a pathway. When the abundance of a protein is increased, this may lead to increased expression of another protein as part of a regulatory feedback loop. This can help maintain homeostasis or control the intensity of a biological response. These correlations can represent amplification or reinforcement mechanisms in biological pathways as well. When one protein's expression increases, it may trigger a cascade of events leading to the augmentation of other proteins, ultimately amplifying the pathway's response. Finally, in some cases, positive correlations may reflect redundancy within a pathway. Multiple proteins within the same pathway may have overlapping functions, and when one is increased another can compensate to ensure that the pathway's function is maintained.

On the other hand, negative correlations may indicate that the proteins have opposing functions or roles within the same biological pathway. One protein might act as an activator while the other acts as an inhibitor. Negative correlations can also suggest regulatory relationships; one protein may directly or indirectly regulate the expression of the other. In some cases, negative correlations can be indicative of compensatory mechanisms; when one protein's expression is reduced, another

protein´s abundance may increase to compensate for the loss of function. This can help maintain overall pathway activity or homeostasis. Negative correlations can also be a sign of complex interactions within a pathway as they often involve multiple feedback loops and intricate regulatory networks. These correlations can reflect the dynamic interplay between various components of the pathway. Finally, negative correlations can highlight the adaptability and plasticity of biological systems. In response to changing environmental or physiological conditions, the expression of proteins may be finely tuned to maintain proper cellular function.

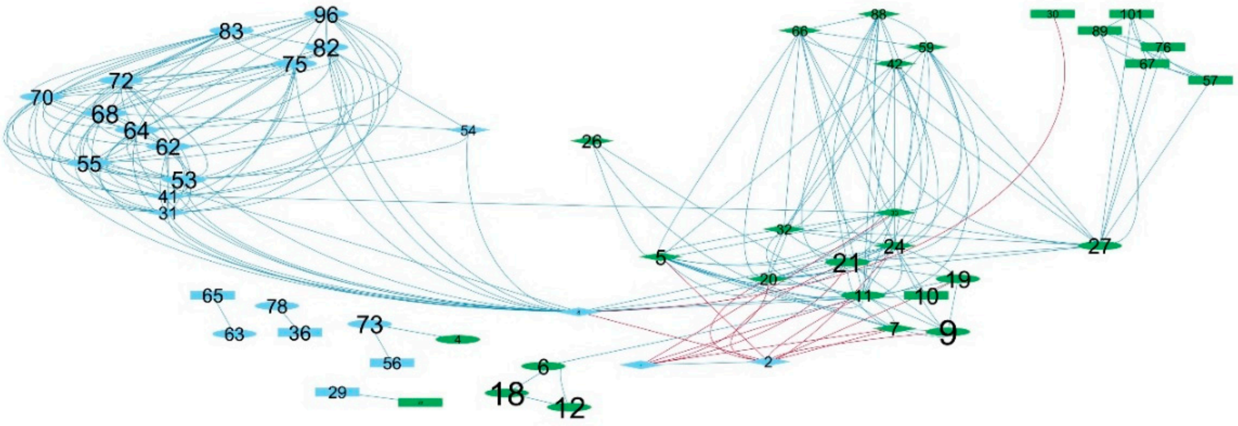

Supplementary Figure S4. Network analysis of proteomic data. Nodes are colored according to their protein levels in permutation univariate tests (shown in blue are proteins which are increased in bleached samples and shown in green are those which are decreased); their shape is related to their significance in uni- or multivariate tests (proteins significant only in the univariate test are shown with diamonds, those only in the multivariate test with squares, and those significant in both analyses with ellipses), and edges are colored according to the sign of the correlations between protein counted data (blue lines represent proteins whose levels are directly proportional and red lines those whose levels are inversely proportional). The size of the number identifying each protein is proportional to the absolute value of its effect size (Table 1).

Supplementary Table S2. Structural characterizing properties of the protein network shown in Supplementary Materials, Figure S4.

**Number of nodes**	**52**
**Number of edges**	219
**Average number of neighbors**	9.953
**Network diameter**	5
**Network radius**	3
**Characteristic path length**	2.480

**Clustering coefficient**	0.823
**Network density**	0.237
**Network heterogeneity**	0.476
**Network centralization**	0.251
**Connected components**	5
