# Supplementary material for: Comparative Proteomic Analysis of Non-Bleached and Bleached Fragments of the Hydrocoral Millepora complanata Reveals Stress Response Signatures Following the 2015–2016 ENSO Event in the Mexican Caribbean
Source: Biology (Basel). 2025 Aug 13;14(8):1042. doi: 10.3390/biology14081042 (PMC12383311; doi:10.3390/biology14081042)
Supplement: Supplementary file 1 [file biology-14-01042-s001.zip › biology-3763541-Supplementary information Figures S1, S2 and S3-edited.pdf]

Comparative proteomic analysis of non-bleached and bleached fragments of the hydrocoral *Millepora complanata* reveals stress response signatures following the 2015–2016 ENSO event in the Mexican Caribbean

Esteban de Jesús Alcántar-Orozco[1], Víctor Hugo Hernández-Elizárraga[1,5], Jesús Eduardo Vega-Tamayo[1], César Ibarra-Alvarado[2], Juan Caballero-Perez[3], Eduardo Rodríguez de San Miguel[4], Alejandra Rojas-Molina[2]

1 Posgrado en Ciencias Químico-Biológicas, Facultad de Química, Universidad Autónoma de Querétaro, Querétaro, México.
2 Laboratorio de Investigación Química y Farmacológica de Productos Naturales, Facultad de Química, Universidad Autónoma de Querétaro, Querétaro, México.
3 Max Planck Institute for Immunobiology and Epigenetics, Freiburg, Germany.
4 Departamento de Química Analítica, Facultad de Química, Universidad Nacional Autónoma de México, Ciudad Universitaria, México City, México.
5 University of Minnesota Genomics Center, Minneapolis, MN, USA.
* Correspondence: Alejandra Rojas-Molina rojasa@uaq.mx

Statistical analysis of spectral count data

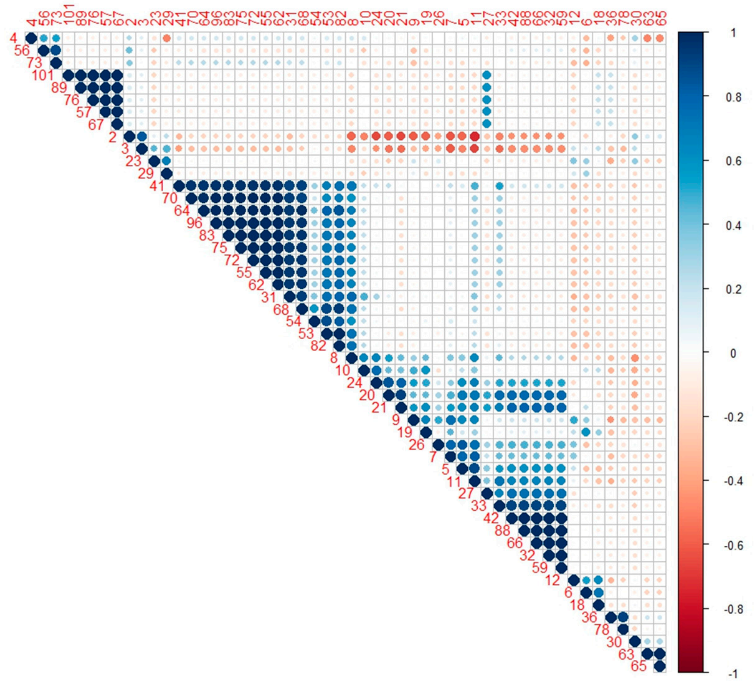

Supplementary Figure S1. Pairwise correlation analysis of the final selected protein expression data. The color bar and the size of the points stand for the magnitude and sign of the correlations.

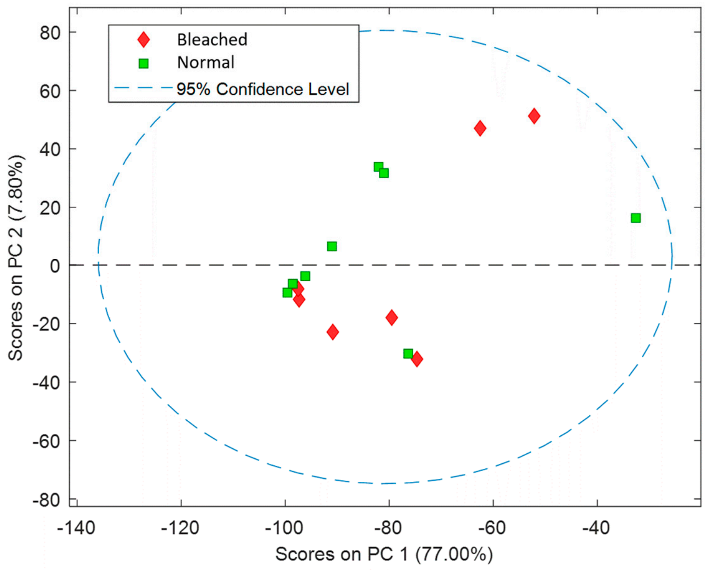

Supplementary Figure S2. Score plot of a two-component PCA model showing both groups of samples.

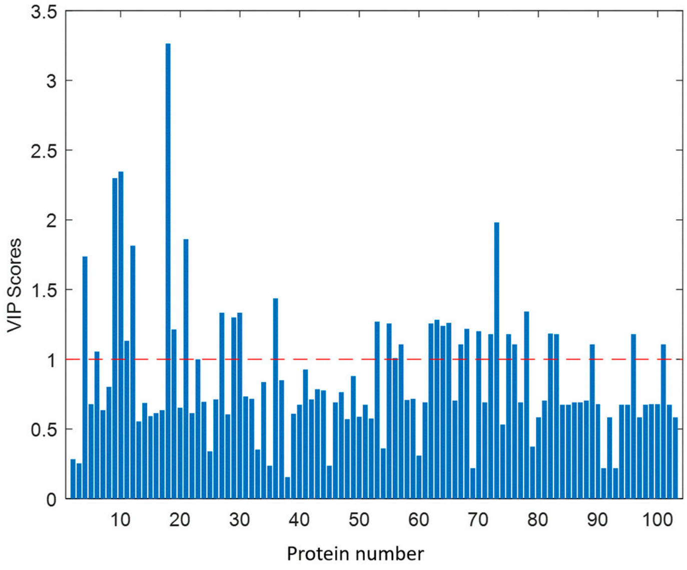

Supplementary Figure S3. VIP score plot for the final classification model in the PLS-DA analysis, where the VIP values for each protein are displayed.
