# Supplementary material for: Comparative Proteomic Analysis of Non-Bleached and Bleached Fragments of the Hydrocoral Millepora complanata Reveals Stress Response Signatures Following the 2015–2016 ENSO Event in the Mexican Caribbean
Source: Biology (Basel). 2025 Aug 13;14(8):1042. doi: 10.3390/biology14081042 (PMC12383311; doi:10.3390/biology14081042)
Supplement: Supplementary file 1 [file biology-14-01042-s001.zip › biology-3763541-Supplementary information Figures S5-S8-edited.pdf]

Comparative proteomic analysis of non-bleached and bleached fragments of the hydrocoral *Millepora complanata* reveals stress response signatures following the 2015–2016 ENSO event in the Mexican Caribbean

Esteban de Jesús Alcántar-Orozco[1], Víctor Hugo Hernández-Elizárraga[1,5], Jesús Eduardo Vega-Tamayo[1], César Ibarra-Alvarado[2], Juan Caballero-Perez[3], Eduardo Rodríguez de San Miguel[4], Alejandra Rojas-Molina[2]

[1] Posgrado en Ciencias Químico-Biológicas, Facultad de Química, Universidad Autónoma de Querétaro, Querétaro, México.
[2] Laboratorio de Investigación Química y Farmacológica de Productos Naturales, Facultad de Química, Universidad Autónoma de Querétaro, Querétaro, México.
[3] Max Planck Institute for Immunobiology and Epigenetics, Freiburg, Germany.
[4] Departamento de Química Analítica, Facultad de Química, Universidad Nacional Autónoma de México, Ciudad Universitaria, México City, México.
[5] University of Minnesota Genomics Center, Minneapolis, MN, USA.
* Correspondence: Alejandra Rojas-Molina rojasa@uaq.mx

Functional annotation and Gene Ontology term assignment

The functional annotation and Gene Ontology (GO) analysis of the 102 proteins provided insights into their biological roles. Figure S5 illustrates the sequence similarity distribution of the identified proteins, measured as the percentage of positive matches along the alignment length. The x-axis represents the percentage of alignment similarity, while the y-axis indicates the number of hits. The distribution shows a strong concentration of high similarity matches.

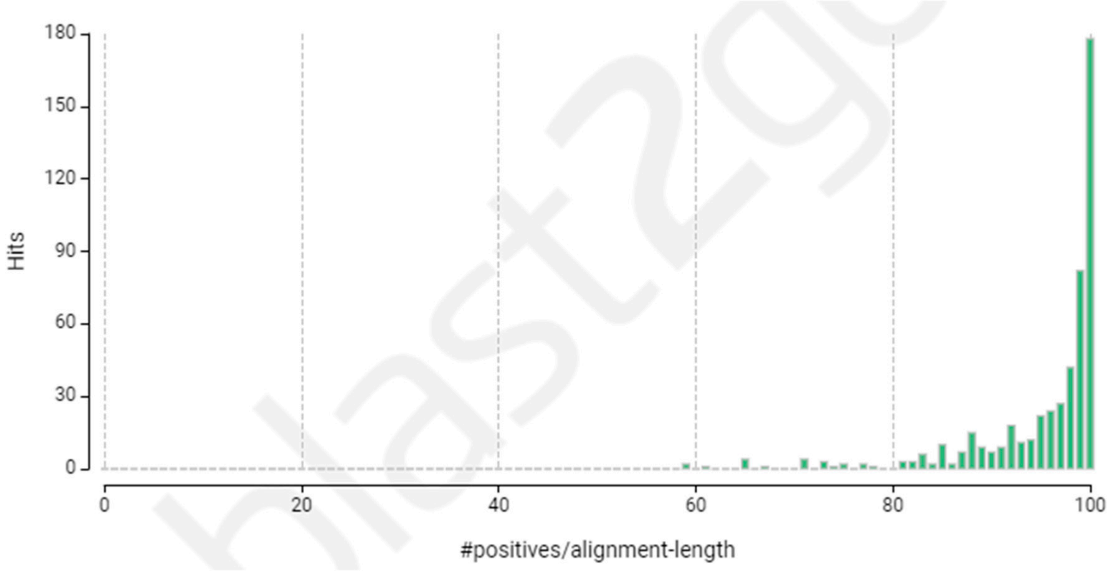

Supplementary Figure S5. Sequence similarity distribution.

Figure S6 presents a hierarchical Gene Ontology (GO) classification for biological processes, illustrating the relationships among functional categories associated with the identified proteins. The analysis highlights key processes such as metabolic regulation, cellular response mechanisms, and macromolecule biosynthesis. Nodes represent different biological processes, with broader categories at the top and more specific functions at the lower levels. The number of sequences mapped to each process is displayed within each node, providing insights into the functional distribution of the dataset. The analysis reveals a strong representation of cellular processes, with a particular emphasis on cell communication and establishment of localization.

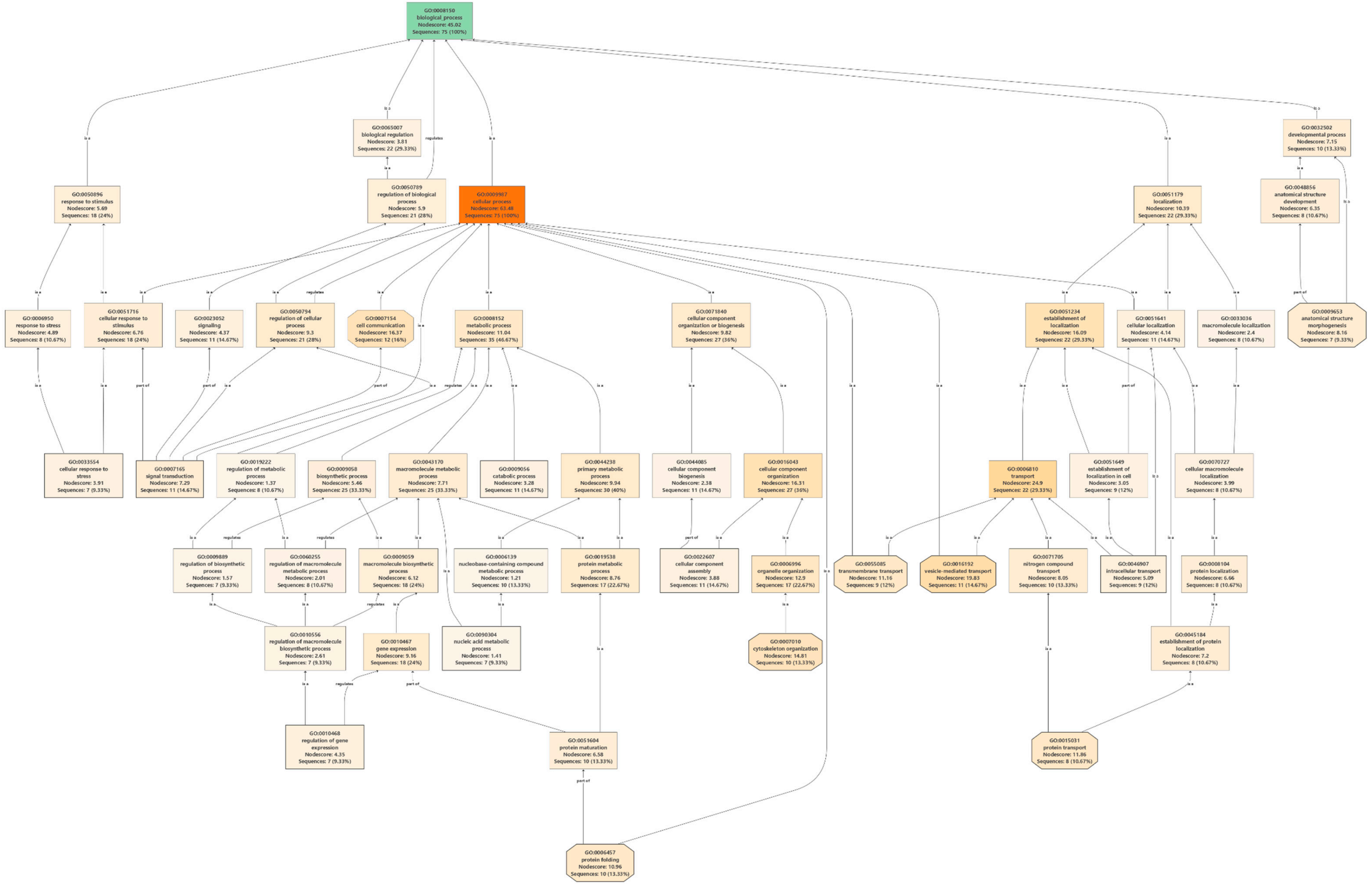

Supplementary Figure S6. Hierarchical GO classification for biological processes.

Figure S7 displays the hierarchical Gene Ontology (GO) classification for molecular functions, highlighting key activities associated with the identified proteins. The analysis reveals a strong representation of binding and catalytic functions, with a particular emphasis on hydrolase and ATPase activities. Several nodes related to binding are also prominent.

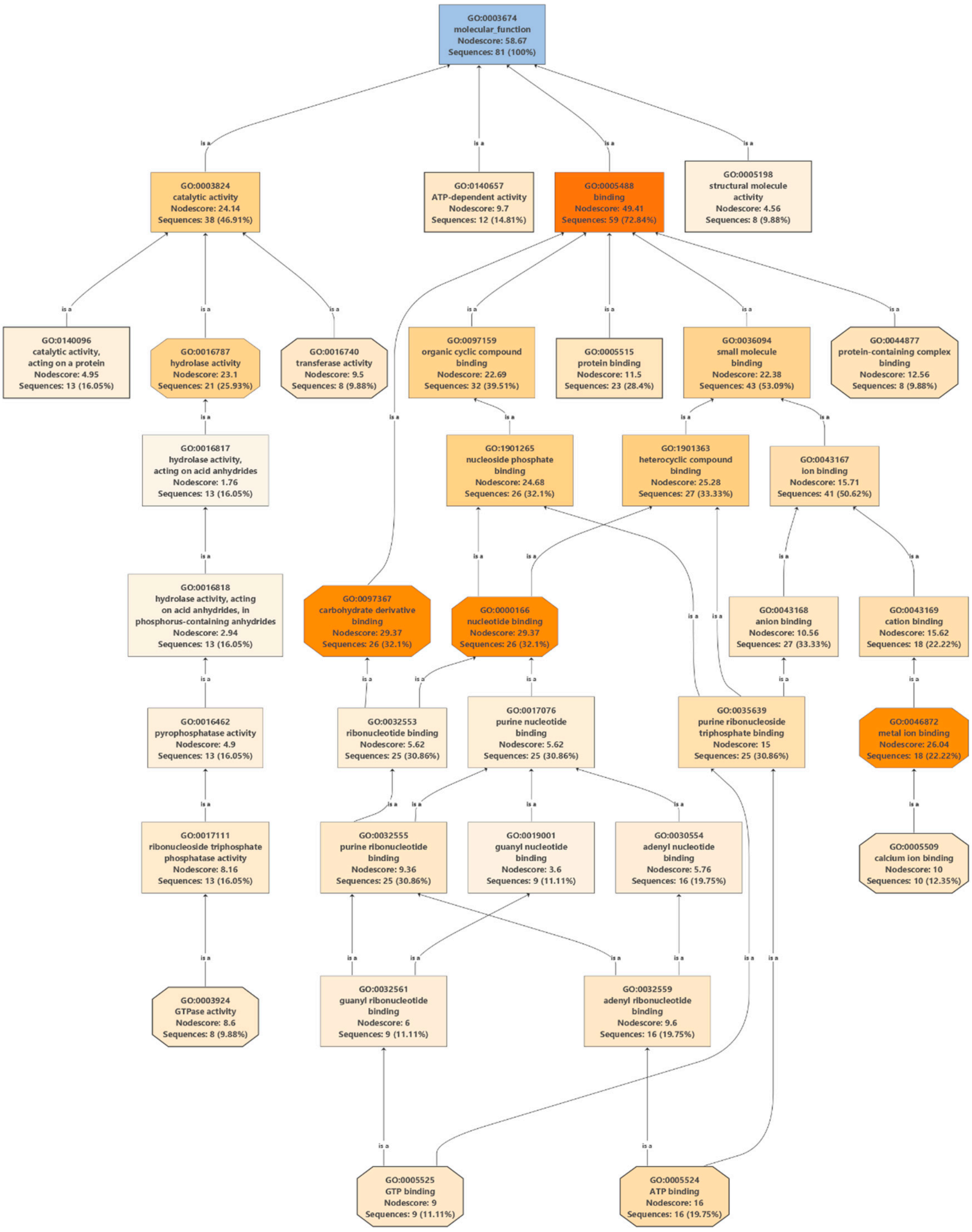

Supplementary Figure S7. Hierarchical GO classification for molecular functions.

Figure S8 presents the hierarchical Gene Ontology (GO) classification for cellular components, highlighting the subcellular localization of the identified proteins. The analysis emphasizes a strong representation of intracellular structures, with proteins mapping primarily to cytoplasmic, membrane-associated, and organelle-related compartments. Notably, a significant proportion of proteins are associated with cellular anatomical structures, organelles, and the cytoskeleton, suggesting functional roles in structural integrity and intracellular transport.

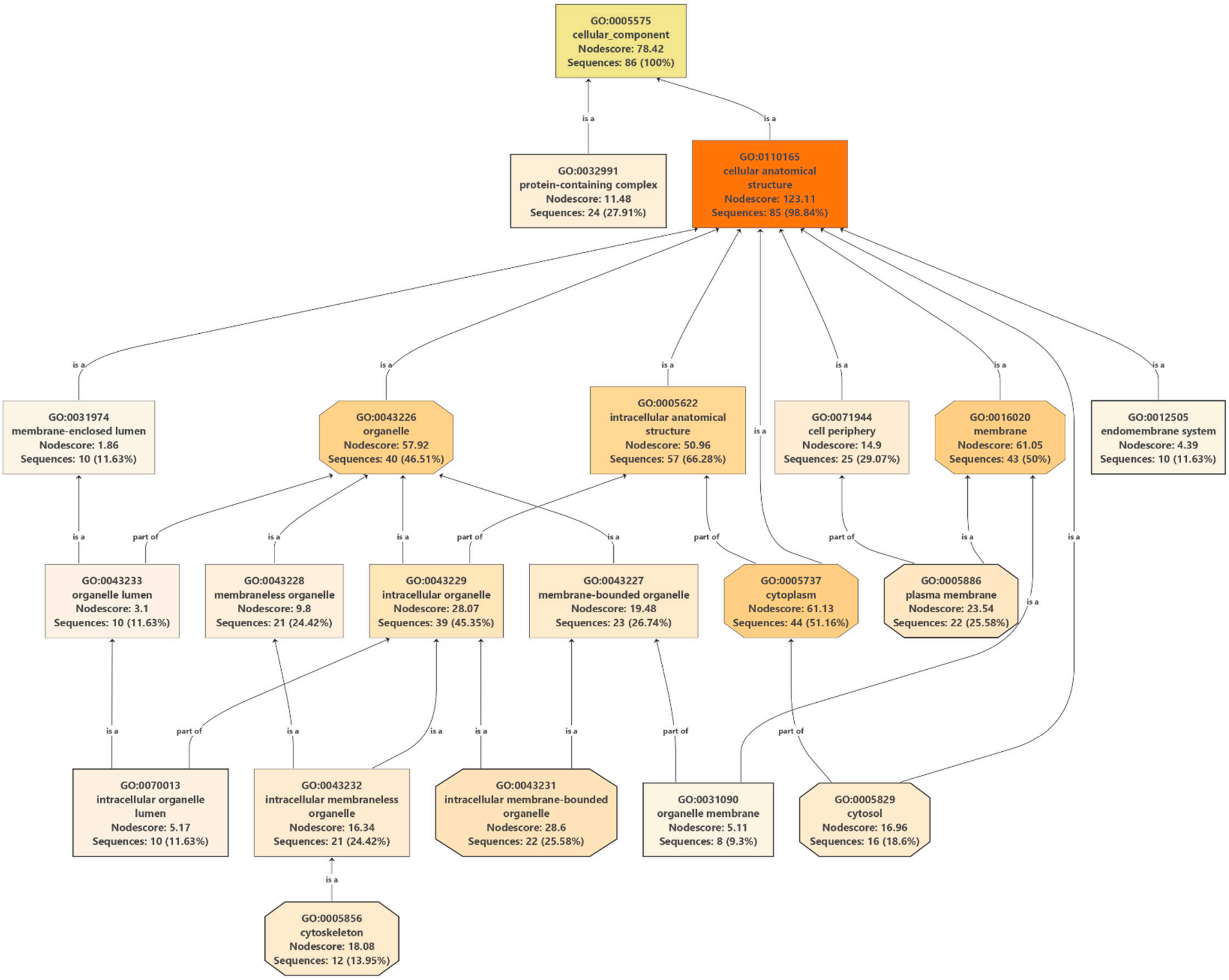

Supplementary Figure S8. Hierarchical GO classification for cellular components.
